# COVID-19 Vaccination, Hospitalization Rates, and Mortality Differ Between People with Diagnosed Immune Mediated Inflammatory Disease and the General Population: A Population-Based Study

**DOI:** 10.3390/vaccines13111130

**Published:** 2025-11-02

**Authors:** Carol A. Hitchon, Carole Taylor, Charles N. Bernstein, Christine A. Peschken, Diane Lacaille, Gilaad G. Kaplan, Jessica Widdifield, Ruth Ann Marrie

**Affiliations:** 1 Department of Internal Medicine, Max Rady College of Medicine, Rady Faculty of Health Sciences, University of Manitoba, Winnipeg, MB R3T 2N2, Canada; 2 Manitoba Centre for Health Policy, Community Health Sciences, Max Rady College of Medicine, Rady Faculty of Health Sciences, University of Manitoba, Winnipeg, MB R3T 2N2, Canada; 3 Arthritis Research Canada and Division of Rheumatology, Department of Medicine, University of British Columbia, Vancouver, BC V5Y 3P2, Canada; 4 Departments of Medicine and Community Health Sciences, University of Calgary, Calgary, AB T2N 1N4, Canada; 5 Sunnybrook Research Institute, ICES, Institute of Health Policy, Management and Evaluation, University of Toronto, Toronto, ON M5S 1A1, Canada; 6 Departments of Medicine and Community Health and Epidemiology, Dalhousie University, Nova Scotia Health, Halifax, NS B3H 1V7, Canada

**Keywords:** COVID-19, vaccine, immune mediated inflammatory disease, rheumatoid arthritis, systemic autoimmune rheumatic disease, inflammatory bowel disease, multiple sclerosis, psoriasis, hospitalization, mortality, administrative health data

## Abstract

Background: Vaccination reduces Coronavirus disease-19 (COVID-19) infection severity. We evaluated COVID-19 vaccine uptake and effectiveness in people with immune mediated inflammatory diseases (pIMIDs) versus the general population. Methods: Using population-based administrative health records, we identified cohorts between 2004 and 2022 with an IMID (rheumatoid arthritis *n* = 10,405, systemic autoimmune rheumatic disease *n* = 5888, inflammatory bowel disease *n* = 7911, multiple sclerosis *n* = 3665, psoriasis *n* = 23,948) who were matched (1:5) by age, sex, and region to general population comparators (*n* = 243,490) without these IMIDs. Between 1 January 2021 and 31 March 2022, rates of COVID-19 vaccine administration, hospitalizations with COVID-19 (Hosp-C), and all-cause mortality were assessed amongst pIMIDs and comparators using multivariable models. Results: More pIMIDs were vaccinated than comparators (87.3% vs. 84.7%, *p* < 0.0001). IMID diagnosis, increasing age, female sex, higher socioeconomic status, urban residence, immunotherapy use, and comorbidities were associated with increased odds of receiving at least two vaccine doses. pIMIDs had higher rates of Hosp-C (79 per 100,000, 95% confidence interval (CI) 77.8–80.2) than comparators (51 per 100,000, 95% CI 50.5–51.3; rate ratio 1.55; 95% CI 1.53, 1.58) and greater mortality [pIMID 1758 deaths, 3.61%; comparators (6346 deaths, 2.61%), RR 1.39 95% CI 1.32, 1.46)]. In multivariable analyses, vaccinated status was associated with less Hosp-C (OR 0.27, CI 0.23, 0.32) and death (HR 0.27 CI 0.24, 0.29); the association did not differ between IMID and comparator groups. Conclusions: Although COVID-19 vaccination reduced the risk of Hosp-C and death in both pIMIDs and comparators, pIMIDs remained at higher risk for both. Since SARS-CoV-2 is now endemic, these findings may inform ongoing vaccination recommendations.

## 1. Introduction

SARS-CoV-2 (COVID-19) vaccination reduces hospitalization, intensive care stays, and mortality related to COVID-19 infection in the general population [1]. In many Canadian provinces, including Manitoba, access to COVID-19 vaccines initially prioritized health care workers, followed by older adults and then the general population. The Manitoba vaccine implementation strategy was partly informed by Manitoba population health data documenting high incidence rates of COVID-19 infection in certain at-risk groups and higher overall provincial COVID-19 infection rates than reported in other Canadian jurisdictions [2]. In Manitoba, immunocompromised patients were not prioritized for vaccination until March 2021, partly due to uncertainty regarding vaccine immunogenicity and safety [3]. We and others have shown that, in people with IMIDs, COVID-19 vaccinations are safe but that some immunotherapies used to treat IMIDs reduce COVID-19 vaccine immunogenicity [3,4,5,6,7]. Thus, a series of at least three COVID-19 vaccines are recommended in immunosuppressed populations [8,9,10]. However, sociodemographic or other factors may have affected COVID-19 vaccine uptake in all groups, including people with IMIDs, thereby augmenting the risk of serious COVID-19 infection in those not vaccinated. Additionally, factors reducing vaccine immunogenicity, such as immunosuppressive use, may augment the risk of serious COVID-19 infection even in those who are vaccinated.

We determined the uptake of COVID-19 vaccines in individuals with a diagnosed IMID across 2021–2022 compared to the general population without these IMIDs. We determined correlates of vaccine uptake. Also, we determined the effectiveness of COVID-19 vaccination in individuals with an IMID and comparators by measuring the association between vaccination status and risk of hospitalizations with COVID-19, all-cause hospitalization, and overall mortality in individuals with IMIDs and comparators.

## 2. Materials and Methods

Study design: This population-based matched cohort study evaluated COVID-19 vaccination status and clinical outcomes (hospitalization with COVID-19, all-cause mortality) occurring from 1 January 2021 to 31 March 2022 among people with and without an IMID residing in the province of Manitoba, Canada.

Data sources: Administrative health records that cover over 95% of the Manitoba population were accessed through the Manitoba Population Research Data Repository at the Manitoba Centre for Health Policy. The databases (and data) used included the Population Registry (dates of birth and death, dates of health care coverage, sex, and region of residence based on the first 3 digits of the postal code); the Hospital Abstracts/Discharge Abstract Database (inpatient hospitalizations, including admission and discharge dates, and up to 25 diagnoses recorded using International Classification of Diseases [ICD] codes, which were the Canadian version of the Tenth Revision [ICD-10-CA]); Medical Services (physician claims, including date of service and 1 ICD-9-CM physician-coded diagnosis); the Drug Program Information Network (DPIN; all community-dispensed prescription medications, including drug name, date of dispensation, and drug identification number [DIN], which is mapped to Anatomical Therapeutic Chemical Classification System codes); the COVID-19 lab testing and results database; and the COVID-19 Vaccinations, Appointments and Screening database (including vaccination date, location of vaccination, and COVID-19 vaccine type). All individual COVID-19 vaccine doses administered in the province are recorded in the latter database. The COVID-19-related ICD10 codes (Supplementary Table S1) are included in the Hospital abstracts/Discharge Abstract and Medical Services databases. These codes enable the identification of hospitalizations where COVID-19 was the diagnosis most responsible for hospitalization. In cases where multiple diagnoses may be classified as “most responsible”, the diagnosis causing the greatest length of stay (LOS) is coded as “most responsible” on the hospital discharge abstract. If the most responsible diagnosis differs from the reason for admission, this is also coded. Data on COVID vaccination is available from Dec 2020 onwards. Data on COVID-19 testing (polymerase chain reaction) is available from Jan 2020 onwards. COVID-19 ICD10 codes were available in 2020 [11]. COVID-19 test positivity was determined during hospital stay (by ICD codes) and the COVID-19 testing and results database. We accessed records from 1 April 2004 to 31 March 2022.

IMID case definitions: We applied validated definitions to identify individuals with a diagnosis of rheumatoid arthritis (RA) [12], inflammatory bowel disease (IBD) [13], multiple sclerosis (MS) [14], psoriasis (PsO) [15], or systemic autoimmune rheumatic disease (SARDS) [16] (Supplemental Table S1) using records from 1 April 2004 to 1 January 2021. All individuals had to have been diagnosed and alive at the study index date (1 January 2021). We chose these IMIDs as they are managed using similar types of immune therapies and have been evaluated in our prior studies of vaccine-mediated immunogenicity [3,4]. Individual IMID patients were matched by age (+/− 1 year), sex, and region of residence based on forward sortation area (first three digits of postal code) to 5 general population comparators without these IMIDs.

Medications: Medications used to treat IMIDs were categorized as immunomodulators, immunosuppressives, or biologics/small molecules (Supplemental Table S2). Medication use was assessed at the study index date (1 January 2021) in IMID and matched populations since most of these medications are not specific for the IMIDs of interest.

Comorbidity: Comorbidity was assessed using a count of Johns Hopkins ACG^®^ System Aggregated Diagnosis Groups (ADGs). We excluded ADG (Time Limited: Major) as we wanted to adjust for chronic disease and excluded ADG (Time Limited: Major-Primary Infections) because infection was an outcome of interest. We also assessed select comorbidities known to be associated with COVID-19 outcomes (diabetes, respiratory disease, and ischemic heart disease) identified using validated definitions [17] (Supplemental Table S1).

Socioeconomic status: Area-level socioeconomic status (SES) was determined by linking an individual’s postal code to dissemination area level data captured in the census by Statistics Canada. We report regional income quintile and socioeconomic factor index version 2 (SEFI) [18]. The SEFI is a regional variable based on a composite of four factors: unemployment rate, average household income, proportion without high school graduation of individuals age 15+ years, and the proportion of single parent households; levels below 0 indicate more favorable SES, and levels above 0 indicate less favorable SES [18].

Location of residence: Residence location was also described by regional health authority and categorized as urban or rural [19].

Vaccination status: We assessed vaccination status using different definitions: any vaccination (1 or more vs. 0) and completion of the primary series of vaccinations (2 or more versus 0). We also assessed the number of COVID-19 vaccines received. We did not account for COVID-19 infection when considering vaccination status as these data were less reliable following Dec 2021, when home administered COVID-19 testing was available and local recommendations were to delay COVID-19 vaccination for 4–6 weeks following COVID-19 infection.

Clinical outcomes: We assessed hospitalizations (any versus none during the study period) with a positive COVID-19 test and all-cause mortality. For comparisons with existing reports, we also assessed all-cause hospitalization (any versus none). During the time frame of this study, all hospitalized patients were tested for COVID-19 at time of admission and/or time of suspicious symptoms or in-hospital contacts. We did not have access to specific cause of death.

### Statistical Analysis

We summarized cohort characteristics using descriptive statistics including frequency (percent [%]) and mean (standard deviation [SD]).

Vaccine uptake: Vaccine uptake over the study period (1 January 2021 to 31 March 2022) was compared between IMIDs and comparators using logistic regression models where the dependent (outcome) variable was (i) any vaccine uptake (1 or more doses versus 0 doses) and (ii) complete vaccine series uptake as defined earlier (2 or more doses versus 0/1 doses) in separate analyses. The primary exposure of interest was the presence of an IMID diagnosis. Following a univariate model including only IMID diagnosis (Model 1), we constructed a model that added demographic characteristics (age, sex, SEFI, urban versus rural residence) (Model 2). The final model (Model 3) also included additional variables for immune therapy use and comorbidity. The primary comorbidity measure used in Model 3 was the number of ADG groups included as a categorical variable (0 [reference], 1, 2, 3, 4, and 5 or more) (Model 3a). Alternate comorbidity measures in sensitivity analyses were (i) the number of ADG groups assessed as a continuous variable (Model 3b) and (ii) specific comorbid conditions previously reported to impact COVID-19 outcomes (Model 3c). We report odds ratios (OR) with 95% confidence intervals (95% CI).

Association between COVID-19 vaccination and hospitalization with COVID-19 and all-cause hospitalization: We used logistic regression models to evaluate the association between COVID-19 vaccination (2 or more doses versus 1 or none) and any hospitalization with COVID-19 during the study period in a sample including both IMIDs and comparators. We also used Poisson regression models to evaluate associations between COVID-19 vaccination and any all-cause hospitalization in IMIDs and comparators. The natural log of person-years was included as the model offset in Poisson models. To calculate person-years, time zero was the index date, which was noted to be 1 January 2021, and the end date was the end of the study, which was noted to be 31 March 2022. Individuals were censored on death or emigration. For both analyses, the initial model included cohort (IMID versus comparators) and vaccination status (Model 1). We added covariates to the models using the same approach as for the logistic models above.

For the logistic regression models, we report odds ratios (OR) with 95% CI. Logistic regression model fit was assessed using the Akaike Information Criterion (AIC—smaller is better), and overall model significance was reported as the chi-square. For the Poisson regression models, we report rate ratios (RR) with 95% CI. Poisson regression model fit is reported as the chi square test based on model deviance.

COVID-19 vaccination and all-cause mortality: We used Cox proportional hazards models to evaluate the association between COVID-19 vaccination (2+ doses versus 1 or none) on all-cause mortality using the same modeling approach as for hospitalizations. Vaccination was included as a time-varying exposure. Schonfelds residuals were used to confirm that the proportional hazards assumptions were met. We report hazard ratios (HR) with 95% CIs.

To test if IMID diagnosis modified the association between vaccination status and hospitalization and mortality outcomes, we included an interaction term (IMID status * vaccination) in all models.

Ethics and funding statement: This study was approved by the Institutional ethics board (HS24647; H2021:055) and the Health Information and Privacy Committee (2021/2022-31). Individual consent was not collected as anonymized administrative health data was used. Cell sizes including less than 6 people are not reported.

Funding was obtained from the Public Health Agency of Canada—COVID-19 Immunity Task Force.

Consumer engagement statement: Consumers were not involved in the conduct of this study.

## 3. Results

We identified 48,698 people with an IMID (RA *n* = 10,405, SARDS *n* = 5888, IBD *n* = 7911, MS *n* = 3665, PsO *n* = 23,948) who were matched to 243,490 comparators. The IMID cohort had a mean (SD) age of 55.65 (16.8) years, and 61.2% were female (Table 1). Most (64%) resided in urban centers and had favorable SFEI scores (mean [SD] −0.13 [0.89]).

Most (85.2%) Manitobans received at least one COVID-19 vaccine. The time to first vaccine was similar for IMIDs and comparators (mean days: IMIDs 482 versus comparators 484).

**Figure 1:**
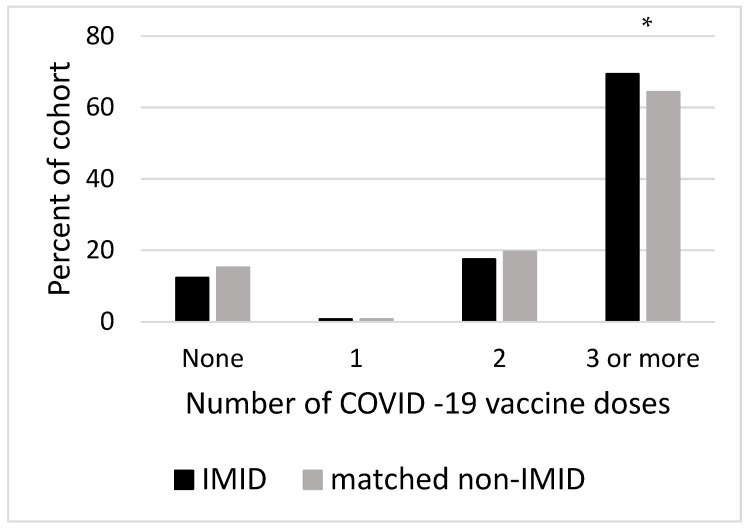
Number of COVID-19 vaccine doses administered. IMID vs. non-IMIDs no vaccine 6006 (12.33%) vs. 37,258 (15.3%), single vaccine 374 (0.77%) vs. 1783 (0.73%); 2 vaccines 8523 (17.5%) vs. 47,766 (19.62%); 3 vaccines 33,795 (69.4%) vs. 156,683 (64.35%); chi-square 207, *p* < 0.0001 (* chi-square IMIDs versus comparators receiving recommended 3 doses 456, *p* < 0.0001). IMID = immune mediated inflammatory disease.

The IMID cohort (87.7%) was more likely than the comparator cohort (84.7%) to have received a least one COVID-19 vaccine (chi-square 283, *p* < 0.001) and had more vaccine doses administered than the comparator cohort (chi-square 207, *p* < 0.0001) [IMID vs. comparators single vaccine 374 (0.77%) vs. 1783 (0.73%); two vaccines 8523 (17.5%) vs. 47,766 (19.62%); three vaccines 33,795 (69.4%) vs. 156,683 (64.35%) chi-square IMIDs versus comparators receiving recommended three doses 456, *p* < 0.0001] (Figure 1).

In adjusted multivariable analysis, the IMID cohort remained more likely to have received a complete vaccine series than the non-IMID comparator cohort (OR 1.27; 1.23, 1.30) (Table 2, see Supplemental Table S3 for full model results). Lower socioeconomic status was associated with 15% reduced odds of receiving a complete vaccine series. Similar findings were seen for receiving at least one (versus no vaccine) (Supplementary Table S4).

Over the study period, the IMID cohort was more likely to be hospitalized with COVID-19 (79 per 100,000 person-years, 95% CI 77.8–80.2) than the comparator cohort (51 per 100,000 person-years, 95% CI 50.5–51.3; rate ratio 1.55; 95% CI 1.53, 1.58). COVID-19 was the diagnosis most responsible for hospitalization for two-thirds of admissions with a positive COVID-19 test (476/706) (67.8), none of which had a different admitting diagnosis. A higher proportion of IMID patients were hospitalized with COVID-19 [159/48,698 (0.33%, 95% CI 0.28, 0.38)] versus comparators [547/243,490 (0.22%, 95% CI 0.21, 0.24), relative risk (RR) 1.45 (95% CI 1.21, 1.73)] even after vaccination with at least two vaccine doses [hospitalization in vaccinated IMID individuals 97/42,318 (0.23%, 95% CI 0.19, 0.28) versus comparators 333/204,449 (0.16%, 95% CI 0.15, 0.18) RR 1.41, 95% CI 1.12, 1.76); hospitalization in unvaccinated IMID individuals 62/6380 (0.97%, 95% CI 0.75, 1.24) versus unvaccinated comparators 214/39,041 (0.55%, 95% CI 0.48,0.63) RR 1.77 (95% CI 1.34, 2.35)]. In unadjusted analyses, the IMID cohort had higher odds of any hospitalization with COVID-19 (OR comparing hospitalization with COVID-19 in IMIDs versus comparators 1.46; 95% CI 1.22, 1.74). In adjusted models, having an IMID diagnosis and not having a complete vaccine series (at least two vaccines) was associated with higher odds of any hospitalization with COVID-19 (Table 3, see Supplemental Table S5 for full model results). There was a dose–response relationship, where a higher burden of comorbidities (number of ADGs) was associated with higher odds of hospitalization with COVID-19. Only 0.4% of hospitalizations with COVID-19 occurred post-COVID-19 vaccination, and all were within 7 weeks of vaccination. Similar findings were observed for all-cause hospitalization (Supplementary Table S6).

A higher proportion of IMID patients died during the study period [1758 deaths in 48,698, 3.61%, 95% CI 3.45, 3.78)] than comparators [(6346 deaths in 243,490, 2.61%, 95% CI 2.54, 2.67), relative risk 1.39 (95% CI 1.32, 1.46)] even after vaccination with at least two vaccine doses [deaths in vaccinated IMID individuals 504/42,318 (1.21%, 95% CI 1.10, 1.31) versus vaccinated comparators 1814/204,449 (0.90%, 95% CI 0.85, 0.94), RR 1.34 (95% CI 1.22, 1.48); deaths in non-vaccinated IMID individuals 1252/6380 (19.62%, 95% CI 18.66, 20.62) versus non-vaccinated comparators 4532/39,041 (11.61%, 95% CI 11.29, 11.93), RR 1.69 (95% CI 1.60, 1.79)]. In unadjusted models, IMID patients had greater hazards of death than the comparator group (HR 1.40, 95% CI 1.33, 1.48), and vaccination reduced hazards of death (HR 0.45, 95% CI 0.42, 0.49). In adjusted models, having an IMID diagnosis and not having a complete vaccine series (at least two vaccines) was associated with higher all-cause mortality (Table 4, see Supplemental Table S7 for full model results).

We did not observe any interaction between vaccine status and IMID status and the outcomes of either COVID-19-related hospitalizations (chi-square 1.6, *p*-value = 0.21) or all-cause mortality (chi-square 0.98, *p*-value = 0.32), indicating that vaccine effectiveness did not differ between people with and without IMIDs.

## 4. Discussion

In this population-based cohort from a Canadian province with universal health care coverage, only 70% of IMIDs received three COVID-19 vaccine doses during the study period. Although people with an IMID were more likely to receive a COVID-19 vaccine during the pandemic compared to people without an IMID diagnosis, people with an IMID were also 55% more likely to have a COVID-19-related hospitalization and had 39% increased overall mortality. COVID-19 vaccination reduced the risk of hospitalization with COVID-19 and death in the IMID and non-IMID cohorts, with no difference in risk reduction observed between the two groups, indicating no difference in vaccine effectiveness between people with or without IMIDs. This suggests that differences in the outcomes reflected underlying differences in these risks between the two cohorts. Other variables associated with an increased risk of adverse outcomes included IMID diagnosis, increased age, male sex, low socioeconomic status, immunomodulatory drug use, and comorbidity burden, which were associated with increased hospitalization with COVID-19 and overall mortality.

Public health strategies for COVID-19 vaccination aim for near universal COVID-19 vaccine coverage, especially for groups at high risk of severe infection and poor outcomes [10,20,21,22]. The World Health Organization initially proposed aiming for 70% COVID-19 vaccination within the population; however, 80–90% may be needed for anti-SARSCoV2 herd immunity, particularly for variants of higher infectious capacity such as Omicron and other emerging variants [23,24]. For the general population, two vaccine doses are needed to develop initial adequate vaccine immunogenicity, and as immunogenicity wanes over time, a “booster” vaccine 6 months following the primary vaccination series is recommended [20]. For people with IMIDs, we and others have shown that a primary vaccine series of at least three doses is required for comparable immunogenicity, in part due to immunosuppressive medications, which adversely impair vaccine responses [3,4,25,26,27,28,29]. In our region, over 83% of the population received at least one vaccine dose, but less than 80% received multiple doses [30]. Although the IMID population did receive more vaccine doses than the population without IMIDs, less than 70% received three vaccine doses during the study period. Similar findings have been reported for IMIDs in other Canadian provinces [31] and globally [32,33]. Consistent with some of these reports, we found lower SES and younger age were associated with lower vaccination rates.

Suboptimal vaccine uptake in IMIDs pre-dates the COVID-19 pandemic [34,35], even in health systems with universal coverage for recommended vaccines. Vaccine hesitancy was reported to be among the top health threats in 2019 by the WHO [36]. Reasons for vaccine hesitancy are complex. For COVID-19 vaccines, these include concerns about vaccine efficacy and safety, gaps in awareness of benefits/risks of vaccination, and sociodemographic factors [37,38]. These concerns were highlighted during the COVID-19 pandemic and seem partly fueled by wide-spread misinformation and conflicting statements from opinion leaders. COVID-19 vaccines are safe for people with IMIDs [39,40]; however, initial uncertainty regarding the safety of these vaccines, particularly for people with autoimmune disorders who were excluded from the first clinical trials, may have contributed to vaccine hesitancy. In Canada and globally, multiple strategies have been proposed to increase COVID-19 vaccine uptake, including information campaigns targeting patients and physicians, strategies to provide easy access to vaccinations, and incentives (i.e., lotteries or monetary payment for being vaccinated) and disincentives (i.e., requirement for vaccines to participate in events) [41,42]. Several of these strategies were initiated in our region [43]. Evidence to support the effectiveness of these measures is mixed. Incentives/disincentives may be beneficial in the short-term but less effective long-term, as recommendations for additional doses change with new variants of concern [41]. Culturally appropriate communication strategies for vaccine counselling, especially for individuals with lower education attainment, reduced household income, rural residence, and without regular health care providers, are needed and should be combined with measures to facilitate access to vaccination, including culturally safe practices and community involvement at vaccination sites [38,44,45,46]. Education that describes the role of vaccines to stimulate protective immunity and how common vaccine side effects can reflect vaccine-mediated immunogenicity when combined with information that promotes the “prosocial” benefits of vaccination for family and society may be more effective than attempts to correct vaccine misinformation [37]. Despite work in this area, vaccine inequity persists [47], and further work is needed to understand the factors that affect vaccine acceptance or hesitancy and how they vary across socioeconomic groups, cultures, and ages.

Our study confirms the efficacy of COVID-19 vaccines in people with IMIDs for reducing COVID-19 hospitalization and overall mortality; however, the rates of these severe COVID-19 outcomes were still higher in people with IMIDs than in people without IMIDs [48,49]. Consistent with our data, other studies have shown that the risk of severe COVID-19 infection in individuals with IMIDs is partly attributed to IMID medication use, disease activity, and, similar to the general population, comorbidities and poor socioeconomic status [50,51,52,53]. Vaccine-mediated immunogenicity wanes over time and is particularly impaired by immunomodulatory drugs, in particular B cell depletors, often used to treat IMIDs [7,54,55,56,57,58,59]. In people with an IMID, reduced post-vaccination immunogenicity associates with higher salivary levels of COVID-19, suggesting a reduced ability to clear the virus when exposed [60]. Other groups have demonstrated that symptomatic breakthrough infections are common in individuals with IMIDs, and although most are mild, infections can be associated with a prolonged symptom duration [61,62,63,64]. In this study, few severe post-vaccination breakthrough infections were observed; we were unable to assess milder infections due to the prevalent use of home-based COVID-19 testing implemented during the study period.

Our study has several strengths. We used population-based administrative health data to compare COVID-19 hospitalizations, all-cause hospitalization, and all-cause mortality in multiple IMIDs (SARDS, RA, IBD, MS, PsO) to those of matched comparators without these diagnoses. We evaluated the efficacy of COVID-19 vaccination as well as the association of immunomodulatory treatment and comorbidities with these outcomes. However, we acknowledge study limitations. The models evaluated hospitalizations associated with a positive COVID test, and for most (67.8%), COVID-19 was also the diagnosis most responsible for admission and the largest contributor to length of hospital stay. For mortality, while the presence of COVID-19 was known, we could not confirm the cause of death, including COVID-19-specific mortality rates, due to delays in data availability. However, all-cause mortality encompasses deaths due to COVID-19 infection and deaths due to other causes, which may have been impacted by COVID-19 infection or pandemic-related reduced access to care. We did not have access to emergency room (ER) visits. In Canada, overall ER visits decreased during times of public health-mandated social distancing and activity restrictions, but patient acuity was high, with increased admission (or death) for COVID-19-related illness particularly for individuals who were older, male, and with comorbidity [65,66]. Reduced vaccine-mediated immunogenicity and increased infection susceptibility resulting from specific IMID disease treatment, in particular corticosteroids and use of B cell-depleting therapies, may have contributed to COVID-19 infection severity. However, the limited sample size and relatively low frequency of some outcomes precluded robust assessments of treatment categories for individual IMIDs as well as comparisons across IMIDs. Administrative health data lack clinical details to determine disease activity and disease specific characteristics, such as IMID-related respiratory, cardiovascular, and thrombotic disease, which can impact clinical outcomes, including COVID-19 infection severity. Similarly, a comparison of different vaccine types was not feasible due to mixed vaccine regimens. However, other data suggest that vaccine efficacy may differ between COVID-19 viral vector vaccines and mRNA vaccines and possibly across COVID-19 mRNA vaccines. The comparator cohort was not a “healthy” cohort but rather a general population cohort without these IMIDs matched by demographics; hence, it included people with non-IMID chronic conditions.

## 5. Conclusions

The SARSCoV2 virus is now endemic, and COVID-19 infection remains a significant health concern. Wastewater surveillance, a strategy used to detect shed virus and predict infection outbreaks, demonstrates moderate seasonal viral activity levels and increasing variants of concern [67]. While we showed that persons with IMID have higher rates of vaccine uptake, certain outcomes are significantly worse. It is unknown how unvaccinated persons with IMIDs do with regards to intensive care unit admissions and COVID-19-related death. Having the vaccine may mitigate against severe outcomes. The potential for severe COVID-19 infection outcomes, increased risk of post-COVID-19 sequalae, and waning vaccine immunogenicity and effectiveness, highlight the importance of continued vigilance and maintaining up-to-date immunizations, particularly for people with IMIDS. Unfortunately, COVID-19 vaccination rates are declining. Public health immunization strategies and advocacy informed by these findings need to be maintained.

## Figures and Tables

**Table 1:** Clinical characteristics of people with diagnosed immune mediated inflammatory disease and matched comparators.

	IMID	Matched Comparators
N	48,698 (16.7)	243,490 (83.3)
RA	10,405 (21.4)	-
SARDs	5888 (12.1)	-
MS	3665 (7.5)	-
IBD	7911 (16.3)	-
PsO	23,948 (49.2)	-
Age (mean (SD))	55.65 (16.8)	55.64 (16.8)
Sex—male	18,907 (38.8)	94,535 (38.8)
Sex—female	29,791 (61.2)	148,955 (61.2)
Regional Health Authority		
IE	5481 (11.3)	27,405 (11.3)
NO	2216 (4.6)	11,080 (4.6)
PT	209 (0.4)	1045 (0.4)
SO	5815 (11.9)	29,075 (11.9)
WE	6000 (12.3)	30,000 12.3)
WP	28,977 (59.5)	144,885 (59.5)
Urban	31,141(64.0)	155,776 (64.0)
Income quintile		
Q1 (lowest)	9573 (19.7)	48,584 (20.0)
Q2	9326 (19.2)	48,005 (19.7)
Q3	9603 (19.7)	48,923 (20.1
Q4	9472 (19.5)	46,828 (19.2)
Q5 (highest)	10,065 (20.7)	48,502 (19.9)
Income quintile missing	659 (1.4)	2648 (1.1)
Socioeconomic factor index-2	−0.13 (0.9)	−0.12 (0.9)
Comorbidity		
Diabetes	7233 (14.9)	33,344 (13.7)
Respiratory	4170 (8.6)	14,704 (6.0)
Ischemic heart disease	6746 (13.9)	25,626 (10.5)
Adjusted Diagnosis Groups (N)		
0	11,878 (24.4)	105,110 (43.2)
1	18,656 (38.3)	89,673 (36.8)
2	13,684 (28.1)	37,980 (15.6)
3	3800 (7.8)	9197 (3.8)
4	624 (1.3)	1424 (0.6)
5 or more	56 (0.1)	112 (0.1)
Mean (SD)	1.24 (0.96)	0.82 (0.88)
IMID medication use (any)	19,923 (40.9)	19,978 (8.2)
Immunomodulators	3090 (15.5)	586(2.9)
Immunosuppressants	9354 (23.4)	1134 (5.7)
Biologics	6170 (31.0)	168 (0.8)
Corticosteroid	10,009(50.2)	19,038 (95.3)

IMID = immune mediated inflammatory disease (comparators matched to IMIDs by age, sex, and region of residence to the general population without these IMIDs), RA = rheumatoid arthritis, SARDS = systemic autoimmune rheumatic disease, MS = multiple sclerosis, IBD = inflammatory bowel disease, PsO = psoriasis, N = number, Regional Health Authority, IE = Interlake-Eastern, NO = Northern, SO = Southern Health-Sante Sud, PT = Public Trustee, WE =Prairie Mountain Health, WP = Winnipeg, SEFI = socioeconomic factor index version 2, Q = quartile, ADG = Johns Hopkins adjusted disease groups adjusted to exclude ADG vector 3 Time Limited Major and ADG vector 4 Time Limited Major-Primary Infections.

**Table 2:** Odds ratios and 95% confidence intervals for variables associated with receiving two or more COVID-19 vaccines.

	Model 1	Model 2	Model 3a	Model 3b	Model 3c
IMID vs. CNT	1.27 1.23, 1.30	1.27 1.24, 1.31	1.06 1.03, 1.10	1.08 1.04, 1.11	1.14 1.10, 1.17

IMID = immune mediated inflammatory disease, CNT = matched comparators. Model 1: IMID diagnosis vs. non-IMID + vaccine (2 doses vs. 1 or 0 doses); Model 2: IMID diagnosis vs. non-IMID + vaccine (2 doses vs. 1 or 0 doses) and demographics (age, sex, SEFI- socioeconomic factor index version 2, urban versus rural residence); Model 3: IMID diagnosis vs. non-IMID + vaccine (2 doses vs. 1 or 0 doses), demographics, IMID medication use, and comorbidity. Separate models assessed comorbidity comparing categories of adjusted ADG (ADG = Johns Hopkins adjusted disease groups adjusted to exclude ADG vector 3 Time Limited Major and ADG vector 4 Time Limited Major-Primary Infections) (Model 3a), total number of ADGs (Model 3b), and specific comorbid conditions previously reported to impact COVID-19 outcomes (diabetes, respiratory disease, and ischemic heart disease) (Model 3c). Policy implication: Vaccination with complete vaccinations more likely with IMID patients even if the rate was overall low.

**Table 3:** Odds ratio and 95% confidence intervals for the association of COVID-19 vaccination and immune mediated inflammatory disease with COVID-19-related hospitalization.

	Model 1	Model 2	Model 3a	Model 3b	Model 3c
IMID vs. CNT	1.54 1.29,1.84	1.46 1.22, 1.75	1.03 0.84, 1.25	1.04 0.85, 1.26	1.19 0.98, 1.44
Vaccine (≥2 vs. 1 or 0)	0.28 0.24, 0.33	0.30 0.26, 0.35	0.27 0.23, 0.32	0.29 0.25, 0.34	0.28 0.24, 0.33

IMID = immune mediated inflammatory disease, CNT = matched comparators. Model 1: IMID diagnosis vs. non-IMID + vaccine (2 doses vs. 1 or 0 doses); Model 2: IMID diagnosis vs. non-IMID + vaccine (2 doses vs. 1 or 0 doses) and demographics (age, sex, SEFI—socioeconomic factor index version 2, urban versus rural residence); Model 3: IMID diagnosis vs. non-IMID + vaccine (2 doses vs. 1 or 0 doses), demographics, IMID medication use, and comorbidity. Separate models assessed comorbidity comparing categories of adjusted ADG (ADG = Johns Hopkins adjusted disease groups adjusted to exclude ADG vector 3 Time Limited Major and ADG vector 4 Time Limited Major-Primary Infections) (Model 3a), total number of ADGs (Model 3b), and specific comorbid conditions previously reported to impact COVID-19 outcomes (diabetes, respiratory disease, and ischemic heart disease) (Model 3c). Policy implication: Vaccination reduces hospitalization with COVID-19 in IMIDs and comparators despite increased hospitalization associated with low socioeconomic status, use of immune medication, and increased comorbidity.

**Table 4:** Hazard ratio and 95% confidence intervals for the association of COVID-19 vaccination and immune mediated inflammatory disease with overall mortality.

	Model 1	Model 2	Model 3a	Model 3b	Model 3c
IMID vs. CNT	1.40 1.33, 1.48	1.43 1.35, 1.51	1.10 1.04, 1.17	1.10 1.04, 1.17	1.27 1.20, 1.35
Vaccine (≥2 vs. 1 or 0)	0.45 0.42, 0.49	0.32 0.30, 0.35	0.27 0.24, 0.29	0.27 0.25, 0.29	0.31 0.28, 0.33

IMID = immune mediated inflammatory disease, CNT = matched comparators. Model 1: IMID diagnosis vs. non-IMID + vaccine (2 doses vs. 1 or 0 doses); Model 2: IMID diagnosis vs. non-IMID + vaccine (2 doses vs. 1 or 0 doses) and demographics (age, sex, SEFI—socioeconomic factor index version 2, urban versus rural residence); Model 3: IMID diagnosis vs. non-IMID + vaccine (2 doses vs. 1 or 0 doses), demographics, IMID medication use, and comorbidity. Separate models assessed comorbidity comparing categories of adjusted ADG (ADG = Johns Hopkins adjusted disease groups adjusted to exclude ADG vector 3 Time Limited Major and ADG vector 4 Time Limited Major-Primary Infections) (Model 3a), total number of ADGs (Model 3b), and specific comorbid conditions previously reported to impact COVID-19 outcomes (diabetes, respiratory disease, and ischemic heart disease) (Model 3c). Policy implication: Vaccination reduces mortality in IMID and comparators despite increased mortality associated with low socioeconomic status, use of immune medication, and increased comorbidity.

## Data Availability

Restrictions apply to the availability of these data. Data from the Manitoba Population Research Data Repository were obtained from the Manitoba Center for Health Policy and are only available following an approved data access application submitted to the Manitoba Center for Health Policy.

## Supplementary Materials

The following supporting information can be downloaded at: https://www.mdpi.com/article/10.3390/vaccines13111130/s1, Table S1: Administrative definitions; Table S2: Categories of immune mediated inflammatory disease medications; Table S3: Odds ratios and 95% confidence intervals for variables associated with receiving two or more COVID-19 vaccines. IMID = immune mediated inflammatory disease, CNT = matched comparators, SEFI = socioeconomic factor index version 2, ADG = Johns Hopkins adjusted disease groups adjusted to exclude ADG vector 3 Time Limited Major and ADG vector 4 Time Limited Major-Primary Infections, AIC = Akaike Information Criterion. Chi-square for overall model significance testing. Model 1: IMID diagnosis vs. non-IMID. Model 2: IMID diagnosis vs. non-IMID and demographics (age, sex, SEFI, urban versus rural residence). Model 3: IMID diagnosis vs. non-IMID, demographics, IMID medication use, and comorbidity. Separate models assessed comorbidity comparing categories of adjusted ADG (Model 3a), total number of ADGs (Model 3b), and specific comorbid conditions previously reported to impact COVID-19 outcomes (Model 3c). Policy implication: Vaccination with complete vaccination series more likely with IMID patients, even if the rate was overall low; Table S4: Uptake of at least 1 vaccine dose in IMIDs compared to CNTS and variables associated with any vaccine uptake (1 or more vs. 0 vaccine doses); IMID = immune mediated inflammatory disease, CNT = matched comparators, SEFI = socioeconomic factor index version 2, ADG = Johns Hopkins adjusted disease groups adjusted to exclude ADG vector 3 Time Limited Major and ADG vector 4 Time Limited Major-Primary Infections. Model 1: IMID diagnosis vs. non-IMID. Model 2: IMID diagnosis vs. non-IMID and demographics (age, sex, SEFI, urban versus rural residence). Model 3: IMID diagnosis vs. non-IMID, demographics, IMID medication use, and comorbidity. Separate models assessed comorbidity comparing categories of adjusted ADG (Model 3a), total number of ADGs (Model 3b), and specific comorbid conditions previously reported to impact COVID-19 outcomes (Model 3c). Policy implication: Vaccination with any vaccination more likely with IMID patients, even if the rate was overall low; Table S5: Odds ratio and 95% confidence intervals for the association of COVID-19 vaccination and immune mediated inflammatory disease with COVID-19 related hospitalization. IMID = immune mediated inflammatory disease, CNT = matched comparators, SEFI = socioeconomic factor index version 2, ADG = Johns Hopkins adjusted disease groups adjusted to exclude ADG vector 3 Time Limited Major and ADG vector 4 Time Limited Major-Primary Infections, AIC = Akaike Information Criterion. Chi-square for overall model significance testing. Model 1: IMID diagnosis vs. non-IMID + vaccine 2 doses vs. 1 or 0 doses. Model 2: IMID diagnosis vs. non-IMID + vaccine (2 doses vs. 1 or 0 doses) and demographics (age, sex, SEFI, urban versus rural residence). Model 3: IMID diagnosis vs. non-IMID + vaccine (2 doses vs. 1 or 0 doses), demographics, IMID medication use, and comorbidity. Separate models assessed comorbidity comparing categories of adjusted ADG (Model 3a), total number of ADGs (Model 3b), and specific comorbid conditions previously reported to impact COVID-19 outcomes (Model 3c). Policy implication: Vaccination reduces hospitalization in IMIDs and comparators despite increased hospitalization associated with low socioeconomic status, use of immune medication, and increased comorbidity; Table S6: Rate ratio and 95% CI for the association of immune mediated inflammatory disease diagnosis and at least two doses of COVID-19 vaccine with all-cause hospitalization. IMID = immune mediated inflammatory disease, SEFI = socioeconomic factor index version 2, ADG = Johns Hopkins adjusted disease groups adjusted to exclude ADG vector 3 Time Limited Major and ADG vector 4 Time Limited Major-Primary Infections. Model 1: IMID diagnosis vs. non-IMID + vaccine 2 doses vs. 1 or 0 doses. Model 2: IMID diagnosis vs. non-IMID + vaccine (2 doses vs. 1 or 0 doses) and demographics (age, sex, SEFI, urban versus rural residence). Model 3-ADG: IMID diagnosis vs. non-IMID + vaccine (2 doses vs. 1 or 0 doses), demographics, IMID medication use, and ADG number versus 0 ADG. Model 3-diagnosis: IMID diagnosis vs. non-IMID + vaccine (2 doses vs. 1 or 0 doses), demographics, IMID medication use, diabetes, respiratory disease, and ischemic heart disease. All estimates chi-squared, *p*-value < 0.0001. All models had adequate goodness of fit reported as Pearson chi-squared based on model deviance. All-cause hospitalization in unvaccinated IMIDs 1727/6380 (27.1%) versus unvaccinated comparators 6518/39,041 (16.7%), relative risk (RR) 1.92, 95% CI 1.83, 2.01. All-cause hospitalization in vaccinated IMID 9709/42,318 (23%) versus vaccinated comparators 33,205/204,449 (16.3%), RR 2.06, 95% CI 2.02, 2.20; Table S7: Hazard ratio and 95% confidence intervals for the association of COVID-19 vaccination and immune mediated inflammatory disease with overall mortality. IMID = immune mediated inflammatory disease, CNT = matched comparators, SEFI = socioeconomic factor index version 2, ADG = Johns Hopkins adjusted disease groups adjusted to exclude ADG vector 3 Time Limited Major and ADG vector 4 Time Limited Major-Primary Infections. Model 1: IMID diagnosis vs. non-IMID + vaccine (2 doses vs. 1 or 0 doses). Model 2: IMID diagnosis vs. non-IMID + vaccine (2 doses vs. 1 or 0 doses) and demographics (age, sex, SEFI, urban versus rural residence). Model 3: IMID diagnosis vs. non-IMID + vaccine (2 doses vs. 1 or 0 doses), demographics, IMID medication use, and comorbidity. Separate models assessed comorbidity comparing categories of adjusted ADG (Model 3a), total number of ADGs (Model 3b), and specific comorbid conditions previously reported to impact COVID-19 outcomes (Model 3c). Policy implication: Vaccination reduces mortality in IMID and comparators despite increased mortality associated with low socioeconomic status, use of immune medication, and increased comorbidity.

## Abbreviations

The following abbreviations are used in this manuscript:

IMID	immune mediated inflammatory disease
RA	rheumatoid arthritis
IBD	inflammatory bowel disease
SARDs	systemic autoimmune rheumatic disease
MS	multiple sclerosis
PS	psoriasis
COVID-19	coronavirus disease of 2019
ICD	International Classification of Diseases
DIN	drug identification number
AGDs	Johns Hopkins ACG^®^ System Aggregated Diagnosis Groups
SES	socioeconomic status
SEFI	socioeconomic factor index version 2
AIC	Akaike Information Criterion
CI	confidence interval
OR	odds ratio
HR	hazards ratio
