# Supplementary material for: COVID-19 Vaccination, Hospitalization Rates, and Mortality Differ Between People with Diagnosed Immune Mediated Inflammatory Disease and the General Population: A Population-Based Study
Source: Vaccines (Basel). 2025 Nov 2;13(11):1130. doi: 10.3390/vaccines13111130 (PMC12656540; doi:10.3390/vaccines13111130)
Supplement: Supplementary file 1 [file vaccines-13-01130-s001.zip › vaccines-3912933-supplementary.pdf]

Supplemental Table 1. Administrative data definitions

Concept Definitions
Rheumatoid arthritis[12] If 2 or more years of data, ≥5 hospitalizations or physician visits or if < 2 years of data ≥3 hospitalization or physician visits with the following ICD codes ICD-9-CM/ICD-10-CA codes 714/M05, M06. For those who were residents for less than 2 years, 3 or more claims sufficed<br>Validation metrics (MCHP and Manitoba clinical cohort): κ = 65.9 [95% CI, 64.1–67.6]; sensitivity, 77.1 [95% CI, 75.4–78.8]; specificity, 90.3 [95% CI, 89.6–91.0]; PPV, 72.0 [95% CI, 70.3–73.8]; NPV, 92.4 [95% CI, 91.8–93.0]; Youden *J* of 67.4.
Systemic autoimmune rheumatic disease[18] If 2 or more years of data, at least 1 hospitalization OR at least 2 physician visits at least 2 months apart within a 2 years span OR at least 1 physician visits by a rheumatologist OR any discharge diagnosis with ICD code 710. (systemic lupus erythematosus, sjogrens, scleroderma (diffuse and limited), polymyositis, and dermatomyositis)<br>Validation metrics: Bayesian hierarchical latent class regression model that does not assume a "gold standard" sensitivity range 60-90 depending on age sex and rural/urban residence
Inflammatory bowel disease[13]<br>If 2 or more years of data, ≥5 hospitalizations or physician visits or if < 2 years of data ≥3 hospitalization or physician visits with the following ICD codes : 555.xx, 556.xx/K50, K51<br>Validation metrics: (MCHP and Manitoba clinical cohort): Crohn's disease sensitivity 89.2 (84.2, 92.8);specificity 89.8 (84.9, 93.3); Youden *J* 0.79; Ulcerative colitis sensitivity 74.4 (67.3, 80.5), specificity 93.7 (89.9, 96.1) Youden *J* 0.68
Multiple sclerosis[15]<br>3 or more hospitalizations, physician visits or medication prescriptions with the following ICD codes 340/ G35 or ATC codes L03AX13, L03AB07, L03AB08, N07XX09, L04AA23, L04AA27, L04AA31, L04AA34<br>Validation metrics: (MCHP and Manitoba clinical cohort) sensitivity 88.6(82.1, 93.3); specificity 58.0 (47.0,68.4); PPV 77(69.7, 83.3); NPV 76.1 (64.1, 85.7)
Psoriasis[16]<br>2 or more years of data and 1 or more hospitalizations, or ≥2 physician visits, or ≥2 medication prescriptions with the following ICD codes 696.0, 696.1, L40.xx, M07.0, M07.2, M07.3 or ATC codes D05AC (antracen derivatives), D05AD (psoralens for topical use), D05AX (other antipsoriatics for topical use), D05BA (psoralens for systematic use), and D05BB (retinoids for treatment of psoriasis).<br>Validation metrics: (MCHP and self-report from people with MS in Manitoba) κ= 0.49 (0.29,0.69),; sensitivity 55.5 (30.7,78.5); specificity 97.3 (95.2, 98.6); PPV 47.6 (25.7,70.2); NPV 98.6 (96.1, 99.1).
Diabetes[11]<br>Over 3 year period with one or hospitalizations or 2 or more physician visits or one or more medication prescriptions with the ICD codes ICD-9-CM code 250 ICD-10-CA codes E10-E14; OR prefix=7 and ICD-9-CM code 250. Exclusions: 1+ prescriptions for metformin (ATC code A10BA) without any other diabetes - related prescriptions, and without a diagnosis for diabetes from a hospital or physician visit.
Respiratory Disease[11]<br>One of the following conditions, one or more hospitalizations with a diagnosis of TRM (using ICD-9-CM or ICD-10-CA codes OR one or more physician visits with a diagnosis of TRM (using ICD-9-CM codes). Codes ICD-9CM 466, 490, 491, 492, 493, 496, ICD-10-CA J20, J21, J40-45
Ischemic heart disease[11]<br>1 or more hospitalizations in 5 yrs OR 2 or more physician visits in 5 years OR 1 physician visit and 2+ prescriptions in 5years. with the following ICD codes ICD-9-CM 410-414, ICD-10-CA 120-122, 124, 125, ATC C01, C07, C08, C09 C10

COVID-19 status for hospitalization[11]<br>U07.1 COVID019 virus identified, U07.3 Multisytem inflammatory syndrome associated with COVID-19 ,
Johns Hopkins ACG® System Aggregated Diagnosis Groups (ADGs) used for comorbidity assessment<br>ADG 9 Likely to Recur: Progressive, ADG 11 Chronic Medical: Unstable, ADG 16 Chronic Specialty: Unstable-Orthopedic, ADG 22 Injuries/Adverse Effects: Major, ADG 25 Psychosocial: Recurrent or Persistent, Unstable<br>ADG 32 Malignancy

Supplemental Table 2. Categories of immune mediated inflammatory disease medications

Disease	Corticosteroids	Immunomodulators	Immunosuppressants	Biologics and small molecules
Rheumatoid Arthritis and Systemic Autoimmune Rheumatic Disease	Prednisone	Cyclosporin<br>Hydroxychloroquine<br>Minocycline<br>Sulfasalazine	Azathioprine<br>Cyclophosphamide<br>Leflunomide<br>Methotrexate<br>Mycophenolate	Infliximab,<br>Adalimumab<br>Etanercept<br>Certolizumab<br>Golimumab<br>Rituximab<br>Belimumab<br>Abatacept<br>Tocilizumab<br>Tofacitinib<br>Baricitinib
Inflammatory Bowel Disease	Prednisone<br>Prednisolone<br>Budesonide<br>Hydrocortisone enema<br>Hydrocortisone acetate<br>Betamethasone	5-ASA (oral, enemas or suppositories)<br>Thiopurines<br>Mesalazine<br>Sulfasalazine<br>Mesalmine<br>Osalazine	Azathioprine<br>6-mercaptopurine<br>Methotrexate	Infliximab<br>Adalimumab<br>Golimumab<br>Ustekinumab<br>Vedolizumab<br>Tofacitinib<br>Ozanimod
Multiple sclerosis	Methylprednisolone<br>Prednisolone<br>Prednisone	Glatiramer acetate<br>interferon-beta 1a<br>interferon-beta 1b<br>dimethyl fumarate<br>Teriflunomide<br>Peg interferon-beta	Azathioprine<br>Methotrexate<br>Mitoxantrone<br>Cyclophosphamide	Natalizumab<br>Fingolimod[2]<br>Alemtuzumab<br>Cladribine<br>Ocrelizumab
Psoriasis		Antracen derivatives (D05AD),<br>other antipsoriatics for topical use (D05AX),<br>psoralens for topical /systemic use	Methotrexate	Etanercept<br>Adalimumab<br>Infliximab<br>Certolizumab pegol<br>Ustekinumab<br>Secukinumab<br>Ixekizumab<br>Guselkumab

Supplemental Table 3. Odds ratios and 95% confidence intervals for variables associated with receiving two or more COVID-19 vaccines.

	**Model 1**	**Model 2**	**Model 3a**	**Model 3b**	**Model 3c**
IMID vs CNT	1.27<br>1.23, 1.30	1.27<br>1.24, 1.31	1.06<br>1.03, 1.10	1.08<br>1.04, 1.11	1.14<br>1.10, 1.17
Sex Male vs female		0.85<br>0.83,0.87	0.87<br>0.85, 0.88	0.85<br>0.84, 0.87	0.85<br>0.84, 0.87
Age [1]		1.01<br>1.01, 1.01	1.00<br>1.002, 1.004	1.01<br>1.00, 1.01	1.01<br>1.01, 1.01
SEFI [1]		0.86<br>0.85, 0.87	0.85<br>0.844 0.86	0.86<br>0.85, 0.87	0.85<br>0.84, 0.86
Urban vs rural		1.41<br>1.38, 1.44	1.40<br>1.37, 1.43	1.40<br>1.38, 1.42	1.42<br>1.39, 1.45
IMID medication			1.30<br>1.25, 1.34	1.31<br>1.27, 1.36	1.45<br>1.39,1.50
Diabetes					1.37<br>1.32, 1.41
Respiratory					1.04<br>0.99, 1.10
Ischemic Heart Disease					0.87<br>0.83, 0.90
ADG 1 vs 0			2.13<br>2.08, 2.19		
ADG 2 vs 0			1.90<br>1.85, 1.97		
ADG 3 vs 0			1.23<br>1.17, 1.30		
ADG 4 vs 0			0.65<br>0.58, 0.72		
ADG 5 vs 0			0.42<br>0.30, 0.59		
Total ADG				1.24<br>1.22, 1.26	
Model Fit<br>AIC<br>Chi-square	 252208<br>265	 247447<br>3580	 242688<br>8082	 246045<br>4813	 246633<br>4304

IMID = immune mediated inflammatory disease, CNT= matched comparators, SEFI = socioeconomic factor index version ADG = John Hopkins adjusted disease groups adjusted to exclude ADG vector 3 Time Limited Major and ADG vector 4 Time Limited Major-Primary Infections. AIC = Akaike Information Criterion. Chi-square for overall model significance testing. Model 1: IMID diagnosis vs non-IMID. Model 2 IMID diagnosis vs non-IMID and demographics (age, sex, SEFI, urban versus rural residence); Model 3 IMID diagnosis vs non-IMID, demographics, IMID medication use and comorbidity]. Separate models assessed comorbidity comparing categories of adjusted ADG (Model 3a), total number of ADGs (Model 3b), and specific comorbid conditions previously reported to impact COVID-19 outcomes

(Model 3c). Policy implication: Vaccination with complete vaccination series more likely with IMID patients even if the rate was overall low

Supplemental Table 4. Odds ratio and 95% confidence limits for the association of immune mediated inflammatory disease and any COVID-19 vaccine uptake (1 or more versus 0).

	**Model 1**	**Model 2**	**Model 3a**	**Model 3b**	**Model 3c**
IMID	1.28<br>1.25-1.32	1.29<br>1.25, 1.33	1.12<br>1.08, 1.15	1.16<br>1.13, 1.20	1.14<br>1.11, 1.18
Sex Male vs Female		0.86<br>0.84, 0.87	0.87<br>0.85, 0.89	0.87<br>0.85, 0.88	0.86<br>0.84, 0.88
Age		1.01<br>1.01, 1.01	1.01<br>1.01, 1.01	1.01<br>1.01, 1.01	1.01<br>1.01, 1.01
SEFI		0.88<br>0.87, 0.89	0.88<br>0.87, 0.89	0.88<br>0.87, 0.89	0.87<br>0.86, 0.88
Urban		1.43<br>1.40,1.46	1.44<br>1.41, 1.47	1.44<br>1.41, 1.47	1.43<br>1.40, 1.47
IMID medication			1.45<br>1.39, 1.46	1.52<br>1.46, 1.57	1.48<br>1.42,1.53
Diabetes					1.38<br>1.34, 1.43
Respiratory					1.05<br>0.99, 1.10
Ischemic heart disease					0.88<br>0.85, 0.92
ADG 1 vs 0			1.42 (1.38, 1.46		
ADG 2 vs 0			1.07<br>1.03, 1.12		
ADG 3 vs 0			0.63<br>0.59, 0.67		
ADG 4 vs 0			0.38<br>0.34, 0.42		
ADG 5 vs 0			0.25<br>0.21, 0.29		
Total ADG				0.94<br>0.93, 0.95	
Model Fit<br>AIC<br>Chi square	 244764<br>282	 240256<br>3423	 238160<br>5376	 239707<br>3911	 239408<br>4173

IMID = immune mediated inflammatory disease, CNT= matched comparators, SEFI = socioeconomic factor index version ADG = John Hopkins adjusted disease groups adjusted to exclude ADG vector 3 Time Limited Major and ADG vector 4 Time Limited Major-Primary Infections. AIC = Akaike Information Criterion. Chi-square for overall model significance testing.Model 1: IMID diagnosis vs non-IMID. Model 2 IMID diagnosis vs non-IMID and demographics (age, sex, SEFI, urban versus rural residence); Model 3 IMID diagnosis vs non-IMID, demographics, IMID medication use and comorbidity]. Separate models assessed comorbidity comparing categories of adjusted ADG (Model 3a), total number of ADGs (Model 3b), and specific comorbid conditions previously reported to impact COVID-19 outcomes (Model 3c). Policy implication: Vaccination with any vaccination more likely with IMID patients even if the rate was overall low

Supplemental Table 5. Odds ratio and 95% confidence intervals for the association of COVID-19 vaccination and immune mediated inflammatory disease with COVID-19 related hospitalization.

	**Model 1**	**Model 2**	**Model 3a**	**Model 3b**	**Model 3c**
IMID vs CNT	1.54<br>1.29,1.84	1.46<br>1.22, 1.75	1.03<br>0.84, 1.25	1.04<br>0.85, 1.26	1.19<br>0.98, 1.44
Vaccine<br>(≥2 vs 1 or 0)	0.28<br>0.24, 0.33	0.30<br>0.26, 0.35	0.27<br>0.23, 0.32	0.29<br>0.25, 0.34	0.28<br>0.24, 0.33
Age		1.04<br>1.04, 1.05	1.03<br>1.02, 1.03	1.03<br>1.02, 1.03	1.03<br>1.03, 1.04
Sex Male vs female		1.06<br>0.91, 1.24	1.05<br>0.90, 1.23	1.03<br>0.89, 1.22	0.990<br>0.847, 1.157
SEFI		1.66<br>1.54, 1.78	1.61<br>1.50, 1.73	1.61<br>1.50, 1.73	1.522<br>1.42, 1.64
Urban vs rural		0.97<br>0.83, 1.13	0.94<br>0.81, 1.10	0.93<br>0.80, 1.09	1.00<br>0.86, 1.17
IMID medication			1.43<br>1.18, 1.75	1.46<br>1.20, 1.77	1.61<br>1.33, 1.95
Diabetes					3.15<br>2.69, 3.70
Respiratory					1.23<br>1.04, 1.61
Ischemic Heart Disease					1.29<br>1.04, 1.61
ADG<br>1 vs 0			2.47<br>1.93,3.15		
ADG<br>2 vs 0			3.65<br>2.82, 4.74		
ADG<br>3 vs 0[2]			4.86<br>3.56, 6.64		
ADG<br>4 vs 0			6.91<br>4.39, 10.89		
ADG<br>5 vs 0			11.35<br>4.00, 32.15		
ADG<br>continuous				1.59<br>1.47, 1.71	
Model Fit<br>AIC<br>Chi-square	 9674<br>285	 8994<br>773	 8819<br>946	 8828<br>986	 8750<br>1059

IMID = immune mediated inflammatory disease, CNT = matched comparators, SEFI = socioeconomic factor index version ADG = John Hopkins adjusted disease groups adjusted to exclude ADG vector 3 Time Limited Major and ADG vector 4 Time Limited Major-Primary Infections. AIC = Akaike Information Criterion. Chi-square for overall model significance testing. Model 1: IMID diagnosis vs non-IMID + vaccine 2 doses vs 1 or 0 doses. Model 2 IMID diagnosis vs non-IMID + vaccine (2 doses vs 1 or 0 doses) and demographics (age, sex, SEFI, urban versus rural residence); Model 3 IMID diagnosis vs non-IMID + vaccine (2 doses vs 1 or 0 doses), demographics, IMID medication use and comorbidity]. Separate models assessed comorbidity comparing categories of adjusted ADG (Model 3a), total number of ADGs (Model 3b), and specific comorbid conditions previously reported to impact COVID-19 outcomes (Model 3c).

Policy implication: Vaccination reduces hospitalization in IMIDs and comparators despite increased hospitalization associated with low socioeconomic status, use of immune medication and increased comorbidity.

Supplemental Table 6. Rate ratio and 95% CI for the association immune mediated inflammatory disease diagnosis and at least two doses of COVID-19 vaccine with all cause hospitalization.

	**Model 1**	**Model 2**	**Model 3-ADG**	**Model 3-Diagnosis**
IMID	1.55<br>1.53, 1.58	1.57<br>1.55, 1.60	1.08<br>1.06, 1.10	1.27<br>1.25, 1.30
Vaccine		0.72<br>0.71, 0.74	0.65<br>0.64, 0.66	0.69<br>0.68, 0.71
Sex (M)		0.95<br>0.94, 0.97	0.95<br>0.93, 0.96	0.92<br>0.91, 0.94
Urban		0.86<br>0.85, 0.87	0.83<br>0.81, 0.84	0.86<br>0.85,0.88
IMID med			1.38<br>1.36, 1.41	1.68<br>1.64,
Diabetes				1.53<br>1.50, 1.56
Respiratory disease				1.06<br>1.03, 1.09
Ischemic heart disease				1.48<br>1.44, 1.51
ADG 1 vs 0			1.95<br>1.91, 2.00	
ADG 2 vs 0			3.52<br>3.43, 3.60	
ADG 3 vs 0			5.70<br>5.53, 5.86	
ADG 4 vs 0			7.95<br>7.57, 8.34	
ADG 5 vs 0			11.43<br>10.06, 13.01	
Model Fit<br>Deviance<br>Chi square	 0.96<br>2.09	 0.91<br>1.83	 0.83<br>1.59	 0.88<br>1.75

IMID = immune mediated inflammatory disease, SEFI = socioeconomic factor index version ADG = John Hopkins adjusted disease groups adjusted to exclude ADG vector 3 Time Limited Major and ADG vector 4 Time Limited Major-Primary Infections. Model 1: IMID diagnosis vs non-IMID + vaccine 2 doses vs 1 or 0 doses. Model 2: IMID diagnosis vs non-IMID + vaccine (2 doses vs 1 or 0 doses) and demographics (age, sex, SEFI, urban versus rural residence). Model 3-ADG: IMID diagnosis vs non-IMID + vaccine (2 doses vs 1 or 0 doses), demographics, IMID medication use and ADG number versus 0 ADG. Model 3-diagnosis: IMID diagnosis vs non-IMID + vaccine (2 doses vs 1 or 0 doses), demographics, IMID medication use diabetes, respiratory disease, ischemic heart disease. All estimates Chi squared p value <0.0001. All models had adequate goodness of fit reported as Pearson Chi-squared based on model deviance. All cause hospitalization in unvaccinated IMIDs 1727/6380 (27.1%) versus unvaccinated comparators 6518/39041 (16.7%), Relative risk (RR) 1.92 95% CI 1.83, 2.01). All cause hospitalization in vaccinated IMID 9709/42318 (23%) versus vaccinated comparators 33205/204449 (16.3%) RR 2.06 95% CI 2.02,2.20). Policy implication: Vaccination reduces all-cause hospitalization in IMIDs and comparators despite increased hospitalization associated with low socioeconomic status, use of immune medication and increased comorbidity.

Supplemental Table 7. Hazzard ratio and 95% confidence intervals for the association of COVID-19 vaccination and immune mediated inflammatory disease with overall mortality.

	**Model 1**	**Model 2**	**Model 3a**	**Model 3b**	**Model 3c**
IMID vs CNT	1.40<br>1.33, 1.48	1.43<br>1.35, 1.51	1.10<br>1.04, 1.17	1.10<br>1.04, 1.17	1.27<br>1.20, 1.35
Vaccine (≥2 vs 1 or 0)	0.45<br>0.42, 0.49	0.32<br>0.30, 0.35	0.27<br>0.24, 0.29	0.27<br>0.25, 0.29	0.31<br>0.28, 0.33
Age		1.11<br>1.11, 1.11	1.09<br>1.09, 1.10	1.09<br>1.09, 1.10	1.10<br>1.10, 1.11
Sex Male vs female		1.46<br>1.40, 1.53	1.36<br>1.30, 1.42	1.36<br>1.30, 1.43	1.36<br>1.30, 1.42
SEFI		1.20<br>1.17, 1.23	1.18<br>1.15, 1.21	1.18<br>1.15, 1.21	1.16<br>1.13, 1.19
Urban vs rural		1.01<br>0.96, 1.06	0.95<br>0.90, 0.99	0.95<br>0.90, 0.99	1.01<br>0.96, 1.06
Diabetes					1.57<br>1.49, 1.64
Resp					1.32<br>1.23, 1.42
IHD					1.49<br>1.40, 1.57
IMID meds			1.16<br>1.09, 1.23	1.61<br>1.10, 1.23	1.29<br>1.22, 1.37
ADG 1 vs 0			1.49<br>1.36, 1.62		
ADG 2 vs 0			2.91<br>2.68, 3.12		
ADG 3 vs 0[2]			5.59<br>5.11, 6.12		
ADG 4 vs 0			9.35<br>8.32, 10.52		
ADG 5 vs 0			12.60<br>9.58, 16.57		
Total ADG				1.81<br>1.77, 1.85	

IMID = immune mediated inflammatory disease, CNT = matched comparators, SEFI = socioeconomic factor index version ADG = John Hopkins adjusted disease groups adjusted to exclude ADG vector 3 Time Limited Major and ADG vector 4 Time Limited Major-Primary Infections. Model 1: IMID diagnosis vs non-IMID + vaccine (2 doses vs 1 or 0 doses). Model 2 IMID diagnosis vs non-IMID + vaccine (2 doses vs 1 or 0 doses) and demographics (age, sex, SEFI, urban versus rural residence); Model 3 IMID diagnosis vs non-IMID + vaccine (2 doses vs 1 or 0 doses), demographics, IMID medication use and comorbidity]. Separate models assessed comorbidity comparing categories of adjusted ADG (Model 3a), total number of ADGs (Model 3b), and specific comorbid conditions previously reported to impact COVID-19 outcomes (Model 3c). Policy implication: Vaccination reduces mortality in IMID and comparators despite increased mortality associated with low socioeconomic status, use of immune medication and increased comorbidity.
